# The Role of Echocardiography Screening at the Stroke Unit

**DOI:** 10.3389/fneur.2020.01003

**Published:** 2020-09-11

**Authors:** Jorge Pagola, Carlos Pagola, Jesús Juega, Teresa González-Alujas, José Alvarez-Sabin, Carlos A. Molina

**Affiliations:** ^1^Stroke Unit, Neurology Department, Vall D'Hebron Hospital, Vall D'Hebron Research Institute, Barcelona, Spain; ^2^Departament de Medicina, Universitat Autònoma Barcelona, Barcelona, Spain; ^3^Cardiology Department, Ciudad de Jaén University Hospital, Jaén, Spain; ^4^Echocardiography Lab Cardiology Department, Vall D'Hebrón Hospital, CIBER-CV, Barcelona, Spain

**Keywords:** echocar diography, strain, PFO, TTE, POCUS, ventricle akinesia, cryptogenic stroke, complex aortic plaque

## Introduction

Cardioembolic stroke is the most disabling cause of stroke and accounts for 30% of ischemic strokes (1, 2). The diagnosis is based on the identification of a potential cardiac source of embolism (SOE) (3). Transthoracic Echocardiogram (TTE) is a non-invasive, available and easy to perform technique to detect SOE with potential therapeutic implications (4, 5). The therapeutic yield of the TTE ranges from 2 to 37.2% depending on the age of the patients and previous heart disease (6, 7). A normal TTE decreases the yield of other advances techniques to detect SOE such as transesophageal echocardiogram (TEE) (8). TTE has been increasingly performed as part of stroke workup in the past few years, whereas TEE is used infrequently as screening test (9). More recently, Point of Care UltraSound (POCUS) concept has increased its field of application and TTE as a screening method at the Stroke Unit is certainly under this rationale (10).

## Subsections Relevant For the Subject

The most frequent SOE are: atrial fibrillation (AF), left ventricle (LV) thrombus, endocarditis, prosthetic valves, and patent foramen ovale (PFO) (11). TTE contributes to the detection of these cardiac SOE as described below:

### TTE—Atrial Fibrillation and Atrial Dysfunction

Every patient who has AF should undergo TTE study to find out the cause of the arrhythmia, which may range from non-cardiac causes (hyperthyroidism, alcoholism) to structural changes involving left atrial (LA) mitral stenosis. TTE will be of use in patients with previous AF and stroke to rule out subjacent valvular disease before the initiation of direct oral anticoagulants. Additionally, in cases of early recurrent embolism whether atrial thrombus is suspected. However, TTE has some difficulty evaluating the LA appendage where a thrombus may be lodged, although the development of real-time three-dimensional TTE can interrogate the LA appendage (12, 13).

It is unsustainable to provide prolonged monitoring to all patients. TTE can help select patients at risk of latent AF by the measurement of atrial dysfunction surrogates. Increased left atrial volume by TTE is one of these markers (14–16). Other TTE parameter related to development of AF is left atrial strain that assesses the left atria distensibility (17).

In recent years, the role of atrial dysfunction and atrial remodeling are being discussed as risk factors for further stroke in patients without AF. Accordingly, patients with severe LA enlargement (indicating atrial remodeling) are at risk of further ischemic stroke despite aspirin (18).

### TTE—Left Ventricular Thrombus and Left Ventricular Dysfunction

The second most prevalent cause of cardioembolic stroke is LV akinesia related or not to LV thrombus (19, 20). TTE will easily detect LV akinesia of cardiac apex and anterior wall with 96% sensitivity and 90% specificity (21). The use of echocontrast can improve the visualization of hyperechogenic mass in at least two orthogonal views of an akinetic area (LV thrombus) (22). In addition, TTE can detect silent ischemic cardiopathy in patients with subclinical coronary disease who will require the attention of cardiologists.

Systolic dysfunction with EF <30% is simply detectable with conventional software of TTE (Simpson formulae) and it is frequently associated with dilated cardiomyopathy (23).

### TTE—PFO and Other Interatrial Changes

In young patients with cryptogenic stroke and a high Risk of Paradoxical Embolism (RoPE) score the presence of a PFO must be evaluated (24). TTE may detects certain normal anatomic variations of cardiac structures that may favor the formation of thrombi (25). The aneurysm of the interatrial septum, defined as >11 mm to >15 mm septum protrusion, is frequently associated with larger PFO (>4 mm size) and increases the risk of further stroke (26, 27). Other structures such as the persistence of the Eustachian valve that directs the blood flow to the PFO are visualized with ultrasound by contrast injection (28).

The Chiari's network is a persistence of a remnant of the atrium formation that has been associated with thrombus creation (29). Finally, in patients with right atrial enlargement, interatrial communication may be suspected (30).

### TTE and Aortic Plaques

Complex aortic plaque is a frequent cause of cryptogenic stroke in older patients (31, 32). Although TEE is the ultrasound modality of choice for the detection of aortic arch atheromatosis, many patients do not tolerate the test or they do not meet the cardiologist's criteria for TEE. Aortic CT is a good alternative but it is not ideal to evaluate mobile thrombi (12). The aortic arch may be rapidly assessed in many patients with TTE from the suprasternal window (33). If a plaque larger than 4 mm size or mobile is detected the TEE can be performed earlier and focused on the area of greatest suspicion.

### TTE and Valvulopathies

The prevalence of mitral stenosis secondary to rheumatic valvulopathies remains a major public health concern in low and middle-income countries (34). However, in our environment rheumatic valvulopathies has decreased dramatically and degenerative mitral stenosis related to older patients is the most common cause. The global prevalence of mitral stenosis has fallen and it is more common to find a patient with a mechanical prosthesis who has a stroke than a patient with classic mitral stenosis. After a stroke the chance of prosthetic valve thrombosis must be assessed. Mobility of the disks and transvalvular gradient could be evaluated by TTE; but in order to rule out non-obstructive thrombosis TEE must be performed. In a different scenario, when a patient with mechanical prosthesis has a cerebral hematoma, TTE may be useful to monitor the functionality of the prosthesis till the anticoagulants are resumed. Degenerative or rheumatic mitral stenosis can be detected with TTE and Doppler gradient by evaluating the thickening and the lack of mobility of the valves. In both native and mechanical valvulopathies, infective endocarditis is a possible cause of stroke. TTE plays a central role in detection of endocarditis; however, when suspected TEE is mandatory to determine the valve involvement and the characteristics of the vegetations (35). Equally, in order to diagnose non-bacterial thrombotic endocarditis (marantic endocarditis) TEE must be performed rather than TTE that may underdetect the vegetations (36).

Valvular and annular calcifications are associated with cover brain infarcts and cognitive decline (37). Some cases of embolization has been observed in mitral caseous degeneration that is detected by TTE as a large echogenic mass with a central echo-lucent area (38). Additionally, detection of aortic stenosis will be important to follow due to its poor prognosis in the long term.

### TTE and Other Infrequent Causes

Cardiac tumors are a classic SOE. However, their frequency is so low (<0.3%) that, in a suspicious image, we must first rule out the possibility of an artifact (some artifacts are only visualized in one plane but not in others) or, more rarely, a thrombus. Both thrombi and tumors have an erratic movement, unlike the cardiac structures (false tendons and trabeculations). When the movement is erratic the study should always be completed with TEE, and in many cases with cardiac MRI. Cardiac tumors most frequently associated with stroke are myxoma and fibroelastoma. Myxoma accounts for more than 50% of tumors (39). Its most common location is in the left atrium, while fibroelastoma is usually located in the aortic valve (40).

If we perform TTE in the first hours after the stroke, we can detect transient apical dyskinesia (Tako-tsubo cardiomyopathy). In order to be distinguished from ventricular akinesia by TTE one should observe a wall motion abnormality not restricted to a single coronary artery territory, affecting the mid-ventricular portions of the anterior, inferior, and lateral walls, in a circumferential configuration (41).

Non-compacted cardiomyopathy is an uncommon cause of stroke that is usually associated with systolic ventricular dysfunction (42). However, in initial stages it can cause embolisms without affecting the systolic function and should be suspected in case of multiple trabeculations with intertrabecular spaces in LV. In this case, once suspicion is made by TTE a cardiac MRI must confirm the diagnosis.

## Point of Care Ultrasound to Assess SOE

In the last years, several protocols are being implemented to enhance the cost-effectiveness of TTE. One approach that will not require any advanced tools is Point of care ultrasound (POCUS). POCUS concept can be applied to the screening method of focused TTE on SOE detection, in neurologist's hands it decreased the length of stay and healthcare costs (43, 44). Indeed, a consensus Document on the recommendations and certifications to perform POCUS for non-cardiologists was published (45). Neurologists have better understood the functioning of cerebral hemodynamics since they have implemented the use of ultrasonography in their clinical practice. Patients admitted to the Stroke Unit often have hemodynamic instability. With POCUS we will evaluate the presence of significant ventricular hypertrophy which will guide us not to hyper hydrate the patient to avoid heart failure. In addition, in those patients in Shock, we can detect the collapse of the inferior vena cava to ascertain if the shock is due to heart failure. Although very rare, we can also detect pericardial effusion or pericardial tamponade in patients with heart failure or in shock.

## Discussion

Stroke Neurologists are experts in ultrasonography field so integrating POCUS into their stroke units makes sense, since 1 out of every 3 patients admitted to the Stroke Unit requires TTE. In regard to the prevalence of cardioembolic sources a rapid screening protocol can be drawn up according to the age of the patient and the presence of vascular risk factors. In TIA patients without vascular risk factors POCUS is less cost-effective. However, we can perform the POCUS in the first 24 h after stroke in order to shorten diagnostic work-up duration to avoid overloading care and hospital stay (essential in times of pandemics).

**Figure 1:**
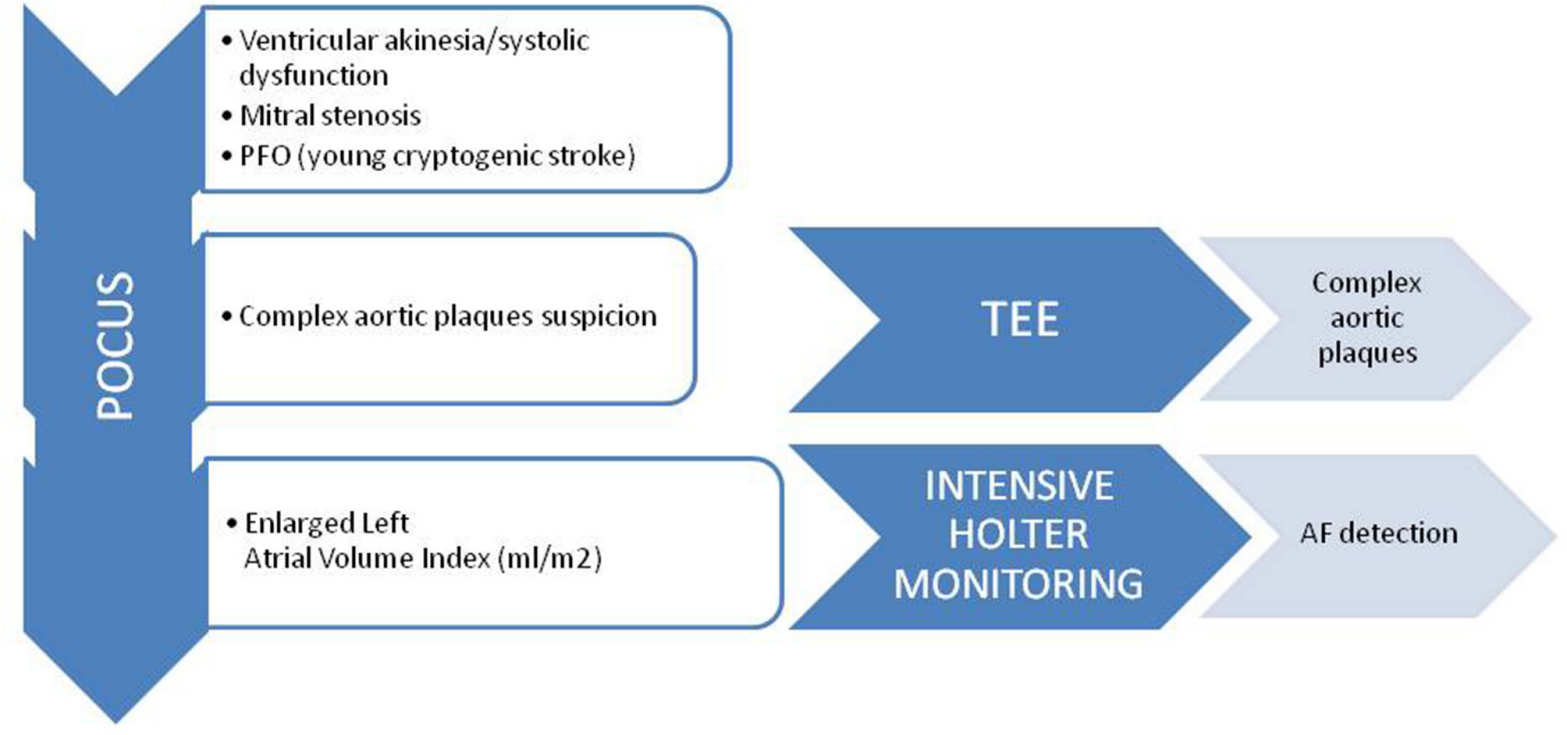
Screening protocol for patients with cardioembolic stroke suspicion. POCUS, point of care ultrasound; TEE, transesophageal echocardiogram.

In the first step of the screening with POCUS (Figure 1), viewing the ventricle, ventricular akinesia and/or severe systolic dysfunction will be detected just checking the movement of the LV. Similarly, mitral stenosis will be assessed by Doppler gradient and mobility of the valves.

If the function of the LV is normal, we will look at the aortic arch as detection of complex aortic plaques is frequent in elderly patients. If we detect a plaque larger than 4 millimeters size, we will request a TEE during admission to evaluate the presence of thrombus related to the complex plaque. Considering that the sensitivity of POCUS is not high, TEE will be required if the suspicion of embolic stroke is still there.

When none of the above provide any data, the size and characteristics of the LA can be checked. It is worth detecting the existence of A-wave pattern to uncover episodes of paroxysmal AF since the A wave is due to atrial contraction. Besides, if the volume of the LA is increased we can indicate intensive ambulatory cardiac monitoring to detect paroxysmal AF. Thus, we will increase the cost-effectiveness of POCUS.

In young patients a saline test with microbubbles can be performed to detect PFO by POCUS. Furthermore, POCUS will allow the evaluation of interatrial septal aneurysm, the Eustachian valve and/or the Chiari network in right atrium.

Other anomalies such as cardiac tumor and mitral stenosis are very rare so we should not base the cost-effectiveness of the POCUS on their detection.

The yield of POCUS to detect SOE increases when patients with large vessel occlusion were examined in the first hours after the stroke onset (46).

The detection of SOE with POCUS has some limitations. Patients with high clinical suspicion for endocarditis (infective or non-bacterial) or mechanical prosthesis should undergo TEE in all cases. In addition, since the evaluation of segmental contractility changes is sometimes challenging, when POCUS is performed by neurologists it is advisable to seek the counsel of a cardiac imaging expert (47).

## Conclusion

TTE remains as a useful technique to screen SOE. POCUS enhances the detection when performed by neurologists at stroke units.

## Disclosure

JP is the President of the Spanish Society of Neurosonology, the National Coordinator of POCUS certification for neurologists in Spain and one of the leaders of the Focused Echocardiography in Neurosonology group of the European Society of Neurosonology and Cerebral Hemodynamics.

## Author Contributions

JP design and conceptualized the manuscript. CP, JJ, TG-A, JA-S, and CM drafted the manuscript. All authors contributed to the article and approved the submitted version.

## Conflict of Interest

The authors declare that the research was conducted in the absence of any commercial or financial relationships that could be construed as a potential conflict of interest.
